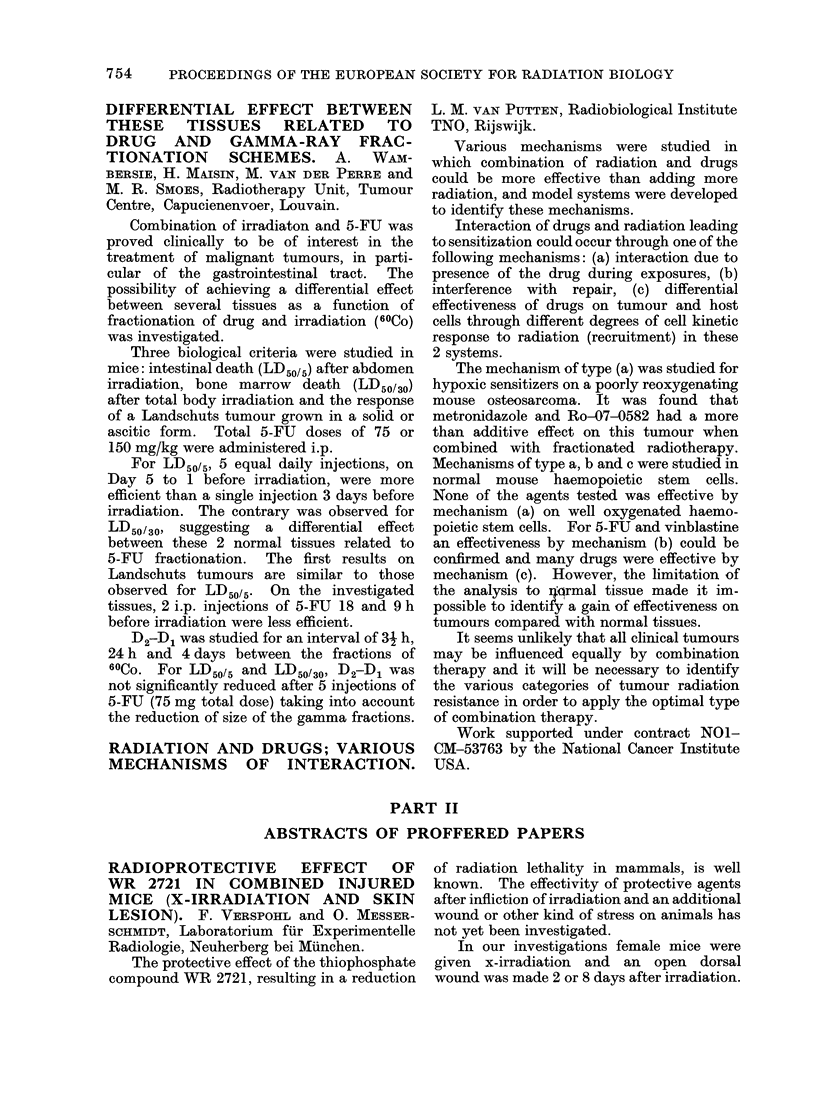# Proceedings: Radiation and drugs; various mechanisms of interactions.

**DOI:** 10.1038/bjc.1975.298

**Published:** 1975-12

**Authors:** L. M. van Putten


**RADIATION AND DRUGS; VARIOUS MECHANISMS OF INTERACTION.** L. M. van Putten, Radiobiological Institute TNO, Rijswijk.

Various mechanisms were studied in which combination of radiation and drugs could be more effective than adding more radiation, and model systems were developed to identify these mechanisms.

Interaction of drugs and radiation leading to sensitization could occur through one of the following mechanisms: (a) interaction due to presence of the drug during exposures, (b) interference with repair, (c) differential effectiveness of drugs on tumour and host cells through different degrees of cell kinetic response to radiation (recruitment) in these 2 systems.

The mechanism of type (a) was studied for hypoxic sensitizers on a poorly reoxygenating mouse osteosarcoma. It was found that metronidazole and Ro–07–0582 had a more than additive effect on this tumour when combined with fractionated radiotherapy. Mechanisms of type a, b and c were studied in normal mouse haemopoietic stem cells. None of the agents tested was effective by mechanism (a) on well oxygenated haemopoietic stem cells. For 5-FU and vinblastine an effectiveness by mechanism (b) could be confirmed and many drugs were effective by mechanism (c). However, the limitation of the analysis to normal tissue made it impossible to identify a gain of effectiveness on tumours compared with normal tissues.

It seems unlikely that all clinical tumours may be influenced equally by combination therapy and it will be necessary to identify the various categories of tumour radiation resistance in order to apply the optimal type of combination therapy.

Work supported under contract NO1–CM–53763 by the National Cancer Institute USA.